# Sequence Evolution and Expression Regulation of Stress-Responsive Genes in Natural Populations of Wild Tomato

**DOI:** 10.1371/journal.pone.0078182

**Published:** 2013-10-18

**Authors:** Iris Fischer, Kim A. Steige, Wolfgang Stephan, Mamadou Mboup

**Affiliations:** Section of Evolutionary Biology, Department of Biology II, University of Munich, Planegg-Martinsried, Germany; Georgia Institute of Technology, United States of America

## Abstract

The wild tomato species *Solanum chilense* and *S. peruvianum* are a valuable non-model system for studying plant adaptation since they grow in diverse environments facing many abiotic constraints. Here we investigate the sequence evolution of regulatory regions of drought and cold responsive genes and their expression regulation. The coding regions of these genes were previously shown to exhibit signatures of positive selection. Expression profiles and sequence evolution of regulatory regions of members of the *Asr* (ABA/water stress/ripening induced) gene family and the dehydrin gene *pLC30-15* were analyzed in wild tomato populations from contrasting environments. For *S. chilense*, we found that *Asr4* and *pLC30-15* appear to respond much faster to drought conditions in accessions from very dry environments than accessions from more mesic locations. Sequence analysis suggests that the promoter of *Asr2* and the downstream region of *pLC30-15* are under positive selection in some local populations of *S. chilense*. By investigating gene expression differences at the population level we provide further support of our previous conclusions that *Asr2*, *Asr4*, and *pLC30-15* are promising candidates for functional studies of adaptation. Our analysis also demonstrates the power of the candidate gene approach in evolutionary biology research and highlights the importance of wild *Solanum* species as a genetic resource for their cultivated relatives.

## Introduction

Numerous efforts have been made in the last decades to understand local adaptation. This phenomenon is defined as the movement of a population towards a phenotype that leads to the highest fitness in a particular environment [1]. As protein divergence alone often cannot explain the phenotypic differences observed between populations/species, gene expression regulation has been suggested to play a key role for many cases of adaptation [2-4]. Modulation of gene expression is crucial for the survival of organisms as environmental changes require fast and specific responses. Experimental evolution studies in microorganisms revealed fast expression divergence between strains of *Saccharomyces cerevisiae* (yeast) [5] and *Escherichia coli* [6] grown in glucose-limited media. In plants, regulatory changes between domesticated crop species and their wild relatives as well as their role in adaptation have been described in *Zea mays* (maize) [7,8] and *Oryza sativa* (rice) [9]. Therefore, one way to investigate local adaptation is to study the expression and regulation of genes that provide a higher fitness under stress conditions. Whole transcriptome analysis has largely been done using microarrays for investigating expression differences in natural populations of model species such as *Drosophila melanogaster* [10,11], to study host shifts in *D. mojavensis* [12] or to analyze invasive plant species such as *Ambrosia artemisiifolia* (common ragweed) [13] and *Cirsium arvense* (Canada thistle) [14]. Recently, sequencing of whole transcriptomes/exomes additionally allowed for large-scale gene expression analysis in different populations or cultivars, e.g. in *D. melanogaster* [15], *D. mojavensis* [16], or *Citrullus lanatus* (watermelon) [17]. However, expression differences have also been analyzed in more detail for particular candidate genes, e.g. cold responsive genes in wild tomato (*Solanum* sp.) [18] or genes involved in root architecture in monkey-flower (*Mimulus guttatus*) [19]. Another question that remains to be answered is to which degree regulatory divergence is adaptive [20]. Most analyses are still focused on the evolution of coding sequences, but examples attributing adaptation to regulatory changes have increased over the last few years [2,21,22].

Terrestrial plants are usually sessile during their life cycle and drought and cold stress are the major abiotic constraints they are facing. Both types of stress have adverse effects on plant growth and crop production [23]. Drought and cold stress lead to accumulation of the phytohormone abscisic acid (ABA), and it has been shown that application of ABA mimics stress conditions [24]. Therefore late embryogenesis abundant (LEA) proteins, which are induced by ABA and were shown to accumulate in vegetative organs during dehydration and low temperature stress [25,26], are good candidates to study adaptation. The LEA proteins are subdivided into seven groups based on their amino acid sequences as well as structural and functional features (e.g. size, hydrophilicity, or glycin content) [27]. In this study we analyze two types of LEA proteins: PLC30-15 [28] encoded by a drought and ABA-inducible dehydrin gene belonging to Group 2, and the ASRs, which belong to Group 7 [27]. Although the functional role of dehydrins still remains speculative, some observations suggest their involvement in abiotic stress tolerance. In *Solanum tuberosum* (potato) and *S. sogarandinum*, dehydrins are drought induced in apical parts and show an increased expression level correlated with cold tolerance in tubers and stems [29]. A previous study of the *pLC30-15* dehydrin revealed that diversifying selection acted on its coding region in a wild tomato population from a dry environment [30]. The other genes used in this study are members of the ABA- and abiotic stress-induced *Asr* (ABA/water stress/ripening induced) gene family [31,32]. ASRs have several functions that help the plant dealing with stress: as monomers with a chaperon function in the cytoplasm [33] or as homo- and heterodimers with DREB (drought response element binding) proteins [34,35] with DNA-binding activity in the nucleus [36]. They also serve as transcription factors associated with the modulation of sugar transport activity [37-39]. Previous studies showed that over-expressed *Asr* genes in transgenic plants lead to higher drought and salt tolerance [40-42]. Using semi-quantitative RT-PCR in cultivated tomato (*S. lycopersicum*), it was demonstrated that *Asr* genes show differences in expression depending on the gene copy or the organ [43]. Analyzing different accessions of wild tomato using Northern Blots, it was shown that *Asr1* and *Asr4* are up-regulated in leaves of plants from humid environments after drought stress [44]. Other studies carried out in wild tomatoes revealed patterns consistent with local adaptation at *Asr* genes in populations that dwell in dry environments [45-47]. These findings make *pLC30-15* and *Asr* genes interesting candidates for studying local adaptation at the gene expression level. 

To understand their role in local adaptation, plants from their native environments are required [48]. For model organisms (e.g. *A. thaliana*, *O. sativa*, or *Z. mays*), an environmental context is not clear and/or cultivation caused reduced diversity due to bottlenecks and artificial selection. Investigating non-model organisms becomes more and more popular, but as they are mostly lacking sequenced genomes it is reasonable to study wild relatives of model organisms [49]. This has successfully been done in relatives of e.g. *A. thaliana* [50-53], *Helianthus annuus* (sunflower) [54,55], *O. sativa* [56], and *S. lycopersicum* [57]. The availability of cultivated tomato genomic resources, the recent divergence of the *Solanum* species, and their clear phenotypic distinction [58] make tomato species a popular plant system that is frequently used to study evolution [57,59]. Most *Solanum* sect. *Lycopersicon* species are native to western South America (Ecuador, Peru, and Chile), along the western and eastern Andean slopes [60]. This study focuses on two recently diverged wild tomato species that show differences in their ecological habitats and features: *Solanum chilense* and *S. peruvianum*. *S. chilense* is distributed from southern Peru to northern Chile where it inhabits arid plains and deserts [58]. It is known to be drought tolerant and can dwell in hyper-arid areas [57,58,61]. Furthermore, it shows a broad range in elevation from sea level up to 3,500 m and therefore experiences large temperature gradients during the year [62]. *S. peruvianum* is distributed from central Peru to northern Chile and inhabits a variety of habitats, from coastal deserts to river valleys [58]. 

At the level of populations, local adaptation can best be studied for organisms with restricted migration [63]. Using the coding sequences, previous population genetic analyses have provided evidence for local adaptation at *Asr2*, *Asr4*, and *pLC30-15* [30,46,47]. Here we sequenced the regulatory regions of these genes from the same populations we had analyzed previously [30,47]. Therefore, we could investigate the evolutionary forces shaping the regulatory regions in direct comparison with the corresponding coding parts of the genes. In addition, we could identify conserved *cis*-acting elements. We also analyzed the expression pattern of *Asr1*, *Asr2*, *Asr4*, and *pLC30-15* in *S. chilense* and *S. peruvianum* accessions that were sampled in close proximity to the populations used for the sequence analysis. We were able to determine differences in gene expression profiles (i.e. intensity and speed) and differences depending on the type of stress or the gene investigated. 

## Materials and Methods

### Sequence analysis: Plant material and sequencing

We sequenced the promoter region of *Asr2* (*pAsr2*), *Asr4* (*pAsr4*), *pLC30-15* (*5’pLC*), and also the downstream region of *pLC30-15* (*3’pLC*). All genes are located on chromosome 4; genomic locations according to the SOL Genomics Network (http://solgenomics.net/) are as follows. *Asr2*: SL2.40ch04:56141779...56142589; *Asr4*: SL2.40ch04:56178656...56180338; *pLC30-15*: SL2.40ch04:63550865...63552237). Two populations from climatically different environments were sampled for each species (Tacna and Quicacha for *S. chilense*; *Tarapaca* and Canta for *S. peruvianum*). Five to seven individuals of each population were analyzed (Table S1 in File S1). A detailed description of these samples is provided in [47,59,64-66]. The *Tarapaca* sample was obtained from the Tomato Genetics Resource Center (TGRC) at the UC Davis (accession number LA2744). The other populations were sampled by T. Städler and T. Marczewski in May 2004 [66,67]. Relevant permits for the collection and import of samples from the Peruvian and Chilean Government were handled by T. Städler and T. Marczewski (as published in [66,67]) or the TGRC. *Solanum ochranthum* (TGRC accession LA2682) was used as outgroup. DNA extraction, PCR amplification, cloning, and sequencing were performed as described in [47]. All primers used in this project can be found in (Tables S2 and S3 in File S1). Sequence alignments are provided in Files S2-S5. Sequence data from this article have been deposited in the EMBL/GenBank Data Libraries under accession numbers HE612885-HE613033.

### Nucleotide diversity analysis, neutrality tests, and haplotype diversity

We measured nucleotide diversity using Watterson’s θ_w_ and Tajima’s π implemented in the DnaSP v5 software [68]. θ_w_ is based on the number of segregating sites [69], and π on the average number of pairwise nucleotide differences among sequences in a sample [70]. We tested for deviations from the standard neutral model using Tajima’s *D* statistic and Fu & Li’s *D* in DnaSP. A significantly negative value of Tajima’s *D* indicates an excess of rare variants as expected under directional selection or population size expansion [71]. A significantly positive Tajima’s *D* value indicates an excess of intermediate-frequency variants as expected under balancing selection or in structured populations [71]. The same is true for Fu and Li’s *D* statistics [72]. Fay & Wu’s *H* was also calculated. A negative Fay and Wu’s *H* indicates an over-representation of high-frequency derived polymorphisms, which is expected under positive selection [73]. A positive Fay and Wu’s *H* indicates an over-representation of intermediate-frequency derived polymorphisms, which is the case if balancing selection was acting [73]. The haplotype test of Depaulis & Veuille [74] was used to assess haplotype diversity (*H*_d_). All neutrality tests were performed using the option Number of Segregating Sites in DnaSP. 

### Motif search in non-coding regions

Motifs in the promoter region were searched using the program PlantCARE [75]. This program contains a database of *cis*-acting regulatory elements and allows for *in silico* analysis of promoter sequences. We limited our search to stress- and hormone-related motifs in conserved regions to provide more information on the kind of stresses that might trigger a response of those genes. We do not describe general transcription factor binding sites like the TATA box.

### Gene expression analysis: Plant material, cultivation and replication

Due to restrictions by the Peruvian government only leaf material was sampled from populations used in the previous sequence analyses [30,47,59,66,67] and the promoter sequence analysis in this work. To perform the gene expression experiments, seeds of six accessions in close proximity to those previously sampled populations were obtained from the TGRC (Table 1). We tested five populations for drought stress and six populations for cold stress (Table 1). As these wild tomato species are outcrossing, we performed cuttings to obtain genetically identical replicates. Tomato seeds of the motherplants were treated with 2.7% NaOCl for 20 minutes to foster germination, and then kept on moistened filter paper at room temperature in the dark until they germinated. The tomato seedlings were then transferred to soil and put into the climate chamber at 22°C with a 16h/8h day/night cycle and 70% humidity. The motherplants were grown until they could provide material for 20-25 cuttings (approximately three months). The cuttings were treated with the Neudofix rooting enhancer (Neudorff, Emmerthal, Germany) and transferred to pots containing soil and vermiculite on top (to ensure nutrition and ventilation). The cuttings were grown for five weeks under the same conditions as the motherplants until they grew roots and three fresh leaves. The *Asr* genes and *pLC30-15* were also sequenced in the motherplants to determine their haplotypes as described above. The experiment was designed as follows. Gene expression was measured at five time points after drought and cold stresses for five and six populations, respectively (Table 1). For each population, three biological and three technical replicates were used (for all time points). 

**Table 1:** Location and habitat characteristics of the accessions used for the expression analysis.

Accession^a^	Species^b^	Nearby population^c^	Collection site^b^	Latitude / Longitude^b^	Altitude^b^ [m]	Annual Precipitation^d^ [mm]	Precipitation wettest month^d^ [mm]	Mean annual temperature^d^ [°C]		Stresses tested
LA1938 (QUI)	*S. chilense*	Quicacha	Quebrada Salsipuedes, *Arequipa*, Peru	15°41' S / 73°50' W	1400	61	31	15.5		drought + cold
LA1967 (TAC1)	*S. chilense*	Tacna	Pachia, Tacna, Peru	17°55' S / 70°09' W	1000	15	5	16.7		drought + cold
LA1969 (TAC2)	*S. chilense*	Tacna	Estique Pampa, Tacna, Peru	17°32' S / 70°02' W	3250	15	5	16.7		cold
LA2744 (TAR1)	*S. peruvianum*	*Tarapaca*	Sobraya, *Tarapaca*, Chile	18°33' S / 70°09' W	400	5	1	17.9		drought + cold
LA2745 (TAR2)	*S. peruvianum*	*Tarapaca*	Pan de Azucar, *Tarapaca*, Chile	18°35' S / 69°56' W	600	5	1	17.9		drought + cold
LA3636 (CAN)	*S. peruvianum*	Canta	Coayllo, Lima, Peru	12°41' S / 76°24' W	No data available	265	77	14.2		drought + cold

a Tomato Genetics Resource Center (TGRC) accession number

b According to TGRC database

c Nearby populations sampled by T. Städler and T. Marczewski, 2004

d Data extracted from WorldClim database (www.worldclim.org); precipitation driest month is always 0 mm

### Stress treatment, RNA extraction and cDNA synthesis

Drought stress was applied by removing the tomato plants from the pots, carefully rinsing and drying their roots and transferring them into a climate chamber at 22°C (according to [43]). For the cold stress, the plants were transferred to a climate chamber at 4°C. Leaves were immediately frozen in liquid nitrogen at five timepoints: unstressed plants, one hour, three hours, six hours, and 24 hours after stress application. Total RNA was isolated using the RNeasy Plant Mini Kit (Qiagen GmbH, Hilden, Germany). DNA was removed using an on-column DNaseI digestion protocol. The RNA integrity was assessed by gel electrophoresis. A NanoDrop 1000 Spectrophotometer (Peqlab, Erlangen, Germany) was used to quantify the RNA and to assess its quality. Only samples with A_260_/A_280_ and A_260_/A_230_ values between 1.9 and 2.1 were used for further experiments. cDNA was synthesized from 1 µg total RNA using SuperScriptIII reverse transcriptase and RNase inhibitor RNaseOUT (both from Invitrogen, Carlsbad, CA, USA) using oligo(dT_20_) primers. The cDNA was treated with RNaseH (New England Biolabs, Ipswich, MA, USA) to remove remaining RNA. 

### Quantitative real-time PCR

Primers for the quantitative real-time PCR (in the following referred to as qPCR) were designed using NetPrimer (http://www.primierbiosoft.com/netprimer) and PrimerBLAST (http://www.ncbi.nlm.nih.gov). As *Asr3* and *Asr5* cannot be distinguished in their coding region [47] they were excluded from this study. qPCR was carried out using iQ SYBR green on a CFX thermocycler (both BioRad, Hercules, CA, USA). Expression of the target genes was normalized by two constitutively expressed reference genes: *CT189* coding for a 40S ribosomal protein [65] and *TIP4I* which was shown to be a very stable reference gene in tomato [76]. As the efficiency was close to 100% for all runs we applied the 2^-ΔΔCq^ method [77] to derive the relative expression quantity from the measured C_q_-values. Quality control, reference gene stability, transformation to relative quantity, and normalization was carried out using the program qbase^PLUS^ [78]. We performed the qPCR for one gene with all populations and timepoints in one run to rule out inter-run variation. To make the results between the different genes comparable, we performed an inter-run calibration in qbase^PLUS^. As this left us with more than one “true” timepoint 0 (= unstressed plants) for each gene we chose to display the results relative to the average of the whole run. We used the two-sample Wilcoxon (Mann-Whitney-*U*) test to determine whether differences in relative expression between stressed and unstressed plants were significant [79]. Importantly, we only compare timepoints within populations to make inferences on the time of gene up-regulation and the peak of expression. As we observe differences in relative expression between the populations at timepoint 0, we do not make comparisons between populations at the same timepoint as this could be misleading. 

## Results

### Incomplete selective sweep in *Asr2* promoter region in the Quicacha population

We sequenced ~1,500 bp upstream of *Asr2*, *Asr4*, *pLC30-15* as this region should contain most *cis*-regulatory regions (i.e. transcription factor binding sites). Additionally, we sequenced the downstream region of *pLC30-15* (~2,300 bp) to determine how far the signature of positive selection at this gene found by [30] extends. However, we cannot rule out other *cis*- or trans-regulatory regions. We wanted to investigate the evolutionary forces acting on regulatory regions of genes involved in stress response. *pAsr2* shows a low nucleotide diversity, especially at Quicacha, compared to the *Asr2* coding region (Table 2, Figure S1a in File S1). Haplotype diversity is also very low in the Quicacha population (Table 3) and Tajima’s *D* and Fu and Li’s *D* are significantly negative (Table 4, Figure S1b in File S1). Indeed, we found only two *pAsr2* haplotypes at Quicacha that were rather similar to each other, where the minor allele occurred only once (Figure S3 in File S1). This indicates that positive directional selection has been acting in this population at *pAsr2*, causing an incomplete selective sweep [66,80]. 

**Table 2:** Nucleotide diversity of *pAsr2*, *pAsr4*, *5’pLC*, *3’pLC*, and their corresponding genes.

π	*pAsr2*	*Asr2* ^a^	*pAsr4*	*Asr4* ^a^	*5' pLC*	*pLC* ^b^	*3' pLC*
Quicacha	0.004	0.016	0.022	0.009	0.030	0.014	0.023
Tacna	0.015	0.020	0.026	0.015	0.028	0.012	0.016
Canta	0.016	0.020	0.032	0.019	0.044	0.016	0.027
*Tarapaca*	0.015	0.022	0.031	0.021	0.043	0.012	0.022
θ_W_							
Quicacha	0.006	0.014	0.021	0.011	0.026	0.010	0.023
Tacna	0.013	0.022	0.026	0.022	0.034	0.013	0.017
Canta	0.020	0.020	0.035	0.021	0.044	0.016	0.031
*Tarapaca*	0.018	0.021	0.030	0.020	0.041	0.012	0.023

a From [47]

b From [30]

**Table 3:** Haplotype diversity of *pAsr2*, *pAsr4*, *5’pLC*, *3’pLC*, and their corresponding genes.

*H*_d_	*pAsr2*	*Asr2* ^a^	*pAsr4*	*Asr4* ^a^	*5' pLC*	*pLC* ^b^	*3' pLC*
Quicacha	0.286	0.800	0.933	0.600	0.667	0.711	0.889
Tacna	0.933	0.978	0.982	0.982	1.000	0.978	0.982
Canta	0.945	0.933	1.000	0.956	1.000	0.978	0.972
*Tarapaca*	0.964	0.956	0.905	0.933	0.889	0.822	0.889

a From [47]

b From [30]

**Table 4:** Results of the neutrality tests for *pAsr2*, *pAsr4*, *5’pLC*, *3’pLC*, and their corresponding genes.

Tajima's *D*	*pAsr2* ^c^	*Asr2* ^a^	*pAsr4*	*Asr4* ^a^	*5' pLC*	*pLC* ^b^	*3' pLC*
Quicacha	-1.688^*^	0.602	0.356	-0.511	0.887	2.342^*^	0.022
Tacna	1.122	-0.372	0.078	-1.429	-0.811	-0.358	-0.499
Canta	-0.936	-0.121	-0.432	-0.548	0.008	-0.339	-0.700
*Tarapaca*	-0.783	0.356	0.170	0.029	0.137	0.010	-0.144
Fu and Li's *D*							
Quicacha	-1.791^**^	-0.121	0.883	0.382	1.853^**^	1.587^**^	-0.432
Tacna	0.661	0.031	-0.230	-2.326^*^	-1.220	-0.384	0.281
Canta	-1.423	-0.590	-1.179	-0.982	-0.916	-0.673	-1.497
*Tarapaca*	-0.784	0.225	-0.214	-0.223	0.180	0.119	-0.401
Fay and Wu's *H*							
Quicacha	NA	-0.927	-3.556	-8.709	-9.190	-1.067	-2.667
Tacna	NA	5.626	3.200	-5.127	-7.727	1.511	-28.673
Canta	NA	0.444	3.778	1.867	7.911	0.978	8.806
*Tarapaca*	NA	0.000	5.143	0.889	-3.167	2.311	1.511

a From [47]

b From [30]

c No outgroup available. Fu and Li’s D* without outgroup was calculated instead.

NA Not applicable

^*^ *P*<0.05, ^**^ *P*<0.01 (significant results are in bold)

### The evolution of the *Asr4* and the *pLC30-15* regulatory regions

Compared to the *Asr4* coding region, where evidence for local adaptation has been shown in a population from a dry environment [47], nucleotide diversity is higher upstream of the gene (Table 2). Additionally, the low haplotype diversity observed at *Asr4* in the Quicacha population increases at *pAsr4* to almost 1 (Table 3), and no deviation from neutrality can be observed (Table 4). This indicates that the forces acting on the *Asr4* gene (directional selection) are weaker in the promoter region. The relatively low level of haplotype diversity detected at *pLC30-15* in Quicacha can also be found at *5’pLC* (Table 3, Figure S4 in File S1). Additionally, Fay and Wu’s *H* becomes very negative in both Quicacha and Tacna (Table 4), indicating positive (diversifying) selection. Nucleotide diversity is generally higher at *3’pLC* than at the *pLC30-15* gene except in the Tacna population (Table 2, Figure S2a in File S1). In addition, Fay and Wu’s *H* becomes extremely negative at the downstream region of the *pLC30-15* gene, indicating positive selection (Table 4, Figure S2b in File S1).

### Different types of motifs in the regulatory regions of *Asr2*, *Asr4 and pLC30-15*

We analyzed the regulatory regions of *Asr2*, *Asr4*, and *pLC30-15 in silico* to identify *cis*-acting elements (short motifs of 4-10 bases). Here we describe only those motifs related to hormone and stress response, and motifs that lie in conserved regions (i.e. without polymorphism) in the alignment of all sequences of both species (see Table S4 in File S1 for additional information of the described motifs). At *pAsr2* we detected a motif conserved in both species involved in salicylic acid responsiveness (TCA-element). We also discovered one motif conserved in *S. chilense* involved in ethylene (ERE). This motif (conserved in *S. peruvianum*) as well as an abscisic acid responsive element (ABRE) was also found at *pAsr4*. In addition, *pAsr4* contains a conserved auxin responsive element (Aux-RR-core). At *5’pLC* we found five conserved ABRE motifs and a motif involved in methyl jasmonate responsiveness (CGTCA-motif); at 3’pLC conserved ABRE and CGTCA-motifs were detected as well as an AuxRR-core. 

Conserved stress-related regulatory elements at *pAsr2* are involved in anaerobic induction (ARE), drought responsiveness (MBS), and general stress and defense response (TC-rich repeats). At *pAsr4* a conserved stress related motif is ARE. TC-rich repeats and MBS motifs are conserved at *5’pLC* and *3’pLC*. *3’pLC* also contains motifs involved in low-temperature responsiveness (LTR) and heat stress responsiveness (HSE). 

### Expression patterns of *Asr1 and Asr2*

**Figure 1:**
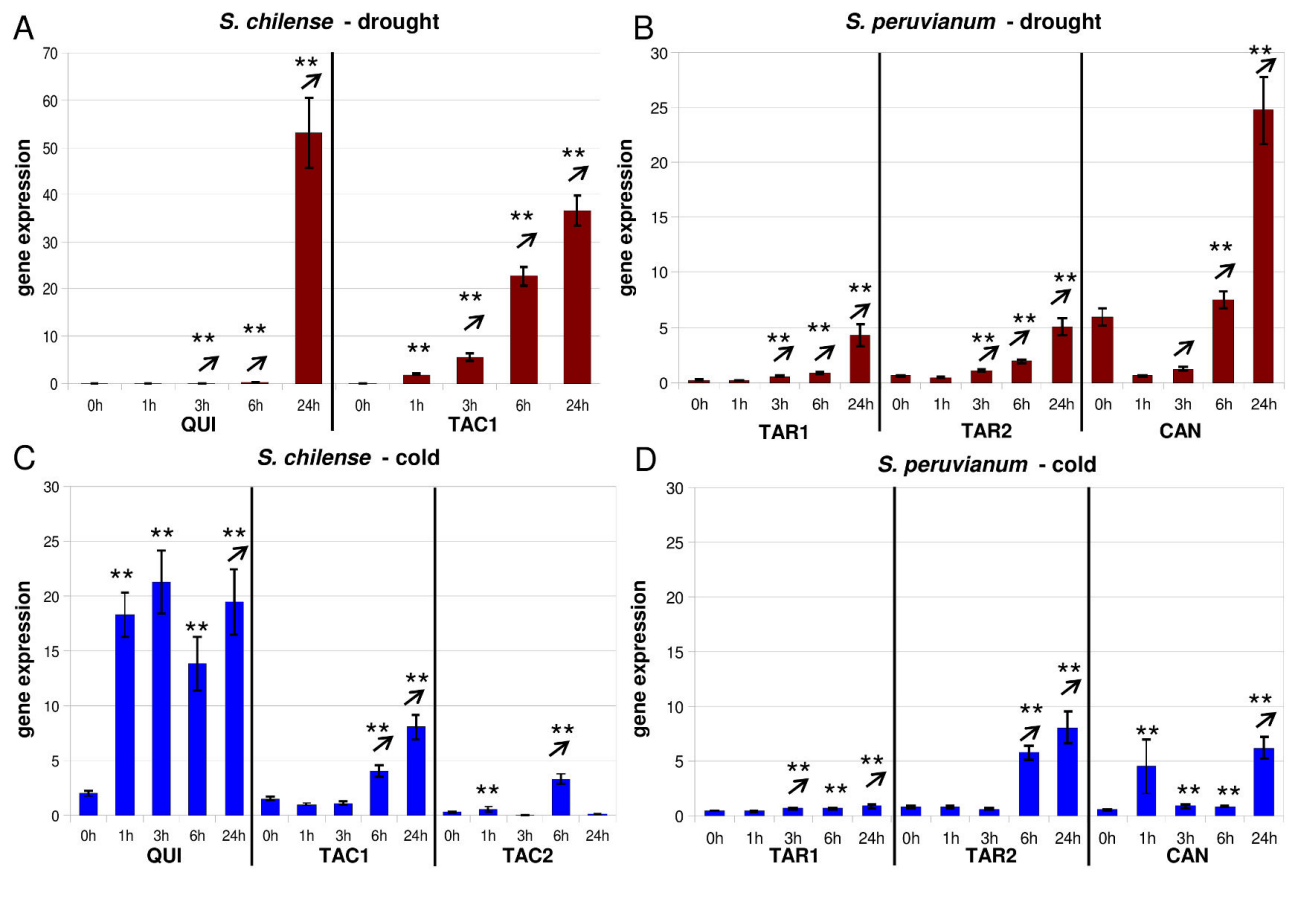
Gene expression of *Asr1* after application of drought and cold stress. Expression is displayed relative to the average of the whole qPCR run in unstressed plants, 1h, 3h, 6h, and 24h after stress application. (A) The following accessions of *S*. *chilense* were measured after drought stress (red bars): LA1938 (QUI) from a dry environment and LA1967 (TAC) from a hyperarid area. (C) The following accessions of *S*. *chilense* were measured after cold stress (blue bars): LA1938 (QUI) from a dry environment and LA1967 (TAC1) from a hyperarid area and LA1969 (TAC2) from a very dry environment and high altitude. The following accessions of *S*. *peruvianum* were measured after (B) drought (red bars) and (D) cold stress (blue bars): LA2744 (TAR1) and LA2745 (TAR2) from a dry environment and LA3636 (CAN) from a humid environment. Vertical lines at bar charts indicate the standard error, asterisks above bar charts indicate significant over-expression compared to the unstressed control (^*^ *P*<0.05; ^**^ *P*<0.01), arrows above bar charts indicate significant over-expression (*P*<0.01) compared to the previous timepoint – meaning the transcript level is increasing.

**Figure 2:**
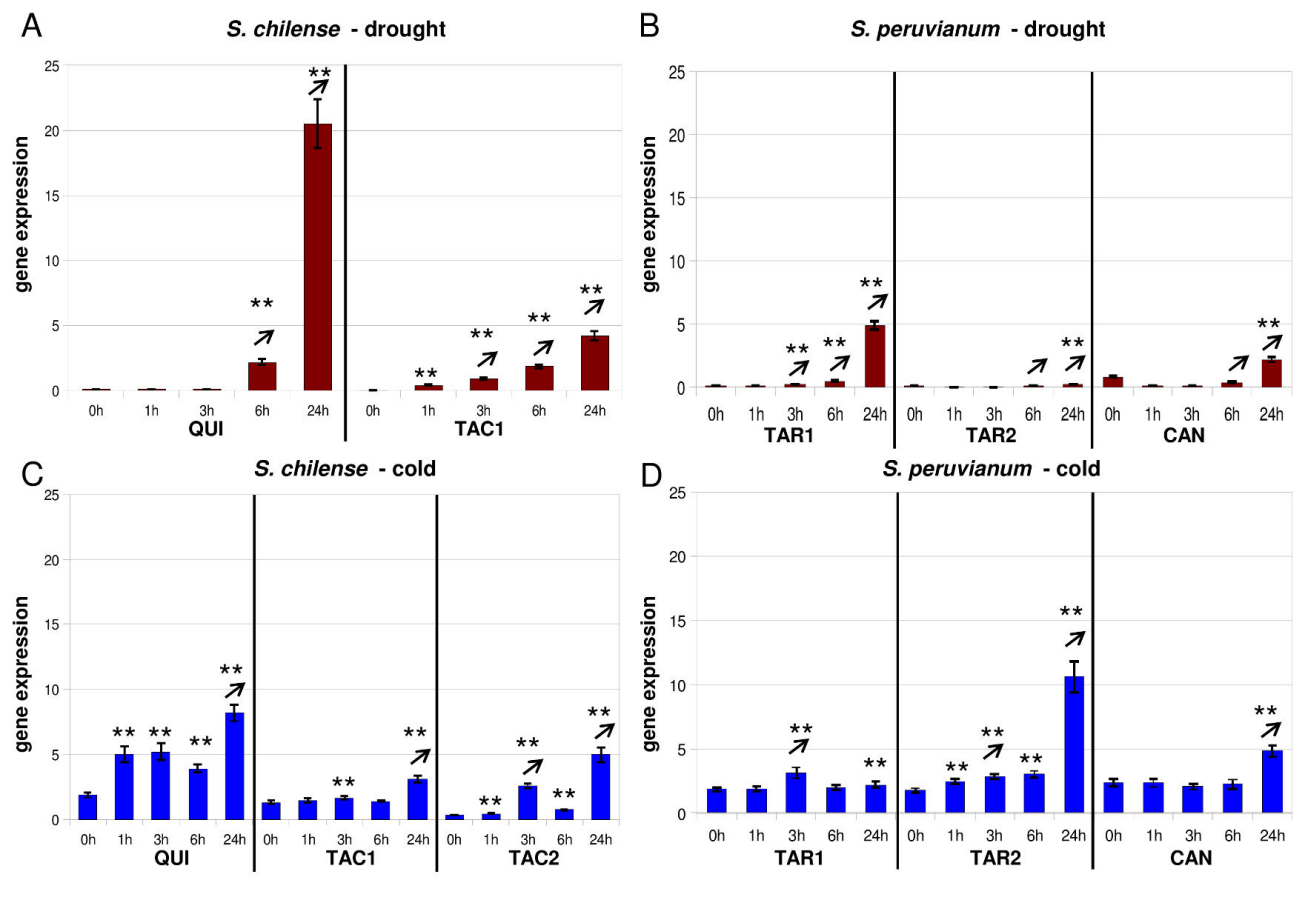
Gene expression of *Asr2* after application of drought and cold stress. (for explanation see Figure 1).

**Figure 3:**
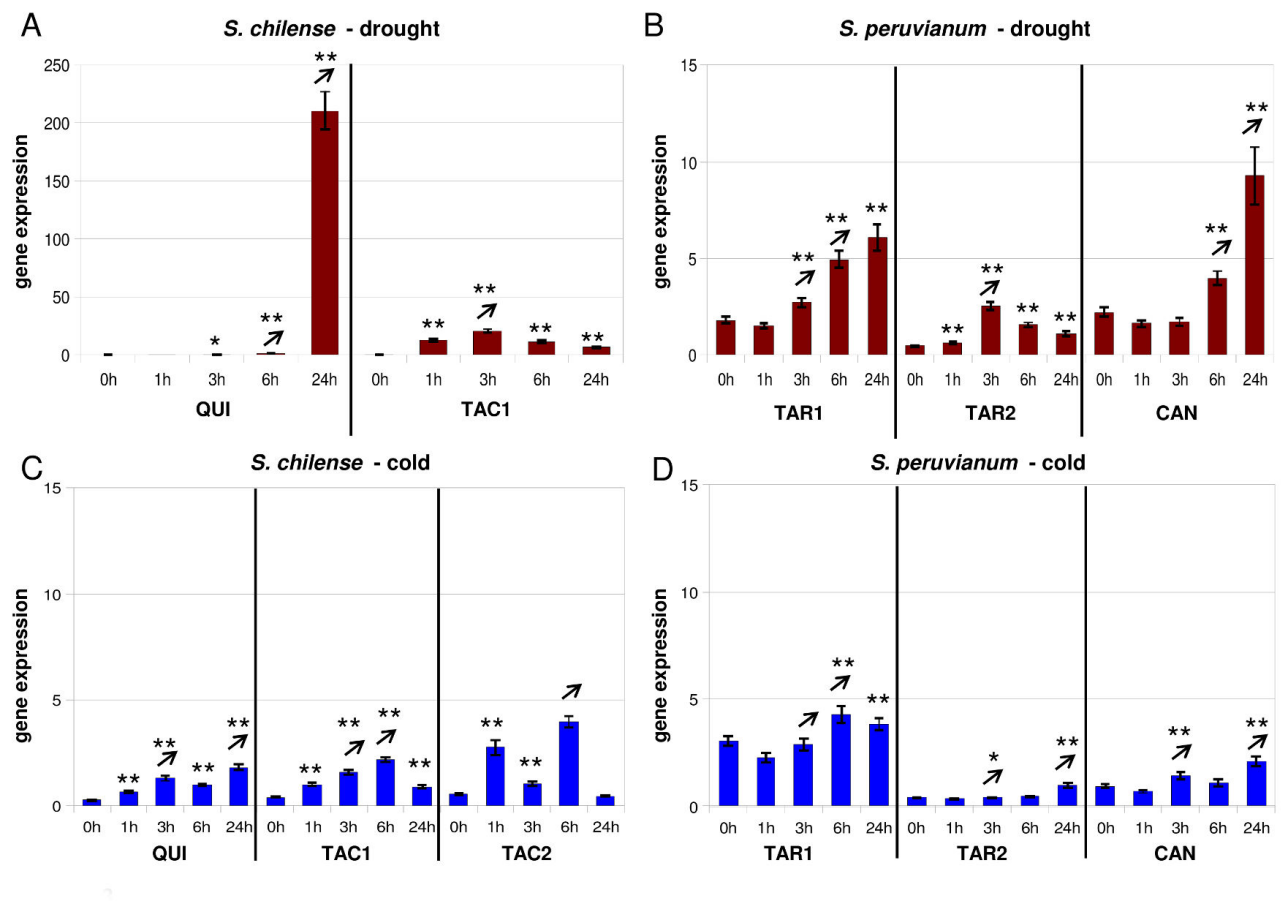
Gene expression of *Asr4* after application of drought and cold stress. (for explanation see Figure 1).

**Figure 4:**
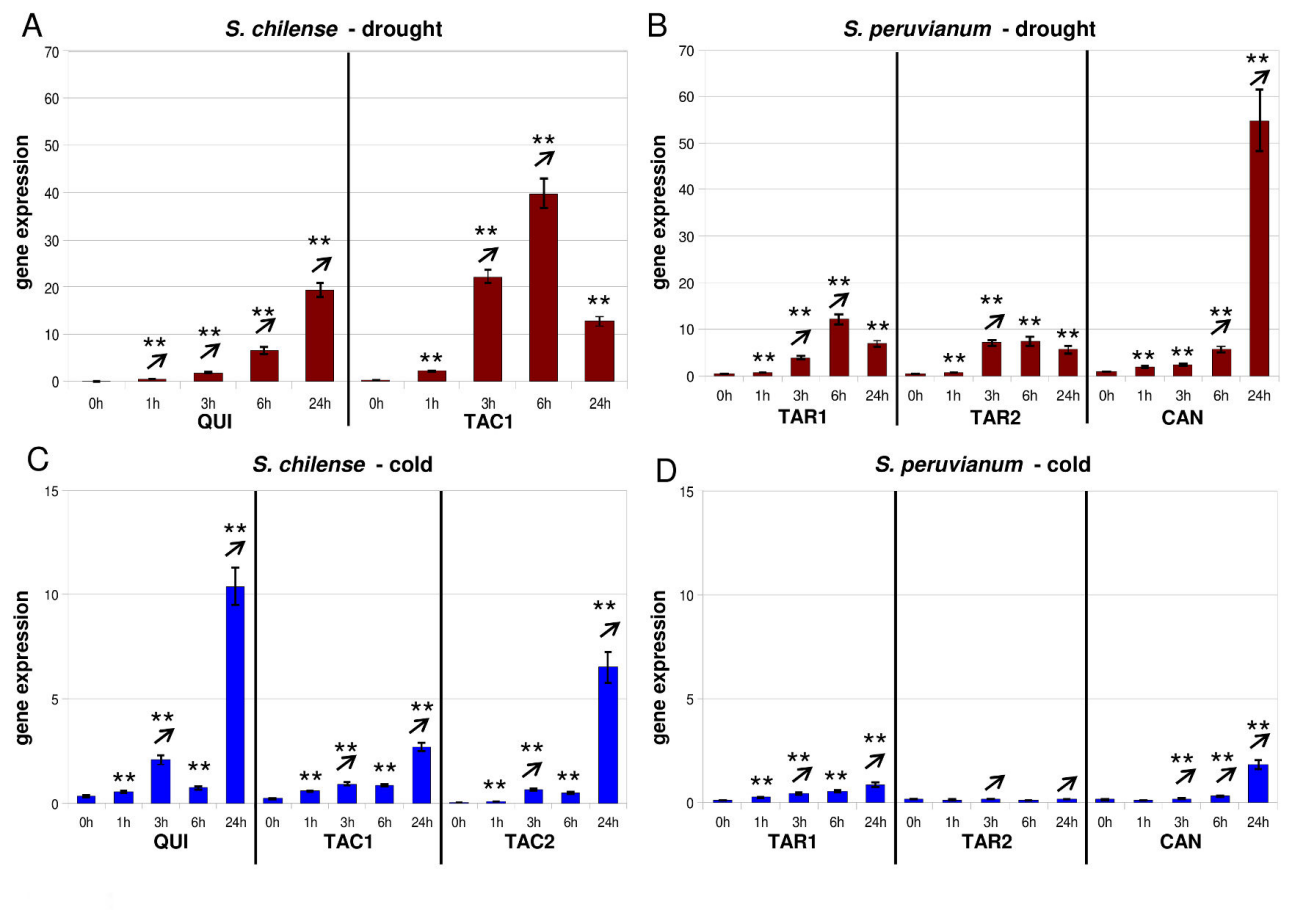
Gene expression of *pLC30-15* after application of drought and cold stress. (for explanation see Figure 1).

We analyzed the expression patterns of *Asr1*, *Asr2*, *Asr4*, and *pLC30-15* in different populations using a time-course experiment after exposing wild tomato plants to cold and drought stress. In general, all genes appear to be more strongly induced in *S. chilense* than in *S. peruvianum* and respond more strongly to water deficit than to cold stress (Figures 1-4). After application of drought stress, *Asr1* is induced after 1-3h in all *S. chilense* and *S. peruvianum* accessions (Figure 1a+b). After cold stress, *Asr1* is induced after 1-6h but at a relatively low level in all *S. chilense* and *S. peruvianum* accessions (Figure 1c+d).

Relative *Asr2* expression is generally quite low compared to the other genes studied here. When applying drought stress to *S. chilense* populations, *Asr2* is significantly up-regulated after 1h in Tacna and 6h in Quicacha (Figure 2a). In *S. peruvianum*, we observe slightly different *Asr2* expression patterns in accessions from the same environment: transcription is induced after 1h in one *Tarapaca* accession (LA2744) and only after 24h in the other *Tarapaca* accession (LA2745) and Canta (Figure 2b). After application of cold stress in *S. chilense*, *Asr2* is significantly up-regulated after 1h in Quicacha and the Tacna accession from a high altitude (LA1969; 3,250m) or after 3h in the Tacna accession from a lower altitude (LA1967; Figure 2c). In *S. peruvianum*, LA2745 shows the highest and fastest (1h) induction of *Asr2* (Figure 2d). LA2744 and the Canta accession are induced more slowly (3h and 24h, respectively; Figure 2d). 

### Faster induction of *Asr4* transcription in a population from a dry environment

In the Quicacha accession of *S. chilense*, *Asr4* is drought induced after 3h and very highly expressed after 24h (Figure 3a). In the Tacna accession from a very dry environment, on the other hand, *Asr4* is already significantly up-regulated after 1h and has its expression peak after 3h (Figure 3a). Based on DNA sequence variation data this population was found to undergo local adaptation and to exhibit signatures of positive selection at the *Asr4* locus [47]. The Tacna accession used in the expression experiments is homozygous for the favored predominant haplotype described in [47]. In *S. peruvianum*, the two *Tarapaca* accessions (both from a similar environment) differ in their expression patterns. In the accession LA2745, *Asr4* is drought induced after 1h and reaches its maximal expression level after 6h (Figure 3b). LA2744, on the other hand, shows a constant increase of *Asr4* transcripts until timepoint 24h (Figure 3b). The Canta accession displays a significant over-expression of *Asr4* after 6h and 24h (Figure 3b). 

*Asr4* induction after cold stress is much lower. All *S. chilense* accessions show a fast induction (1h) of *Asr4* transcripts (Figure 3c). However, the transcript level seems to be higher in the Tacna accession from a high altitude (LA1969) compared to the one from a lower altitude (LA1967; <1,000m) and the accession from Quicacha. In *S. peruvianum*, *Asr4* induction is much slower: after 3h in Canta and 6h in *Tarapaca* (Figure 3d). 

### High expression levels of *pLC30-15* after drought stress

After application of drought stress, *pLC30-15* is induced fast (after 1h in all accessions) and reaches its peak of transcription after 3-6h – except for the Quicacha and Canta accessions where the expression level increases until 24h (Figure 4a,b). Transcript levels appear to be higher in *S. chilense* than in *S. peruvianum* after drought stress, but to a lesser degree than for the *Asr* genes (Figure 4a,b). After cold stress, *pLC30-15* has a lower transcript level in both species (Figure 4c,d). *pLC30-15* is significantly up-regulated after 1h in all *S. chilense* accessions and expression keeps increasing until 24h (Figure 4c). In *S. peruvianum*, we can again observe differences in expression in accessions from the same environment: *pLC30-15* is induced after 1h in LA2744 but no induction can be detected in accession LA2745 (both *Tarapaca* – Figure 4d). In Canta, *pLC30-15* is induced after 3h and increases until 24h (Figure 4d). 

## Discussion

We analyzed the regulatory regions of stress-responsive genes in wild tomato populations from different environments and compared them to their corresponding coding regions, which were previously studied [30,47]. Our most salient observations are as follows. Sequence analyses suggest that the *Asr2* promoter region and the downstream region of *pLC15-30* in *S. chilense* populations from dry environments have been under positive selection. The gene expression experiments suggest that, in general, the genes show a higher induction in *S. chilense* than in *S. peruvianum* and respond more strongly to water deficit than to cold stress. In particular, we found that *pLC30-15* and *Asr4* are highly drought induced in the *S. chilense* population from Tacna (a very dry environment). As these genes also exhibit signatures of positive selection in the coding region [30,47] they may therefore be of potential interest for further functional studies of adaptation. Since we did not perform statistical analysis between populations, however, these observed trends are suggestive and further experiments are needed to verify them. In the following, we discuss these findings in more detail.

### Sequence variation of regulatory regions of stress-responsive genes

Studying *cis*-regulatory elements has been of great interest over the last years as it has been suggested that phenotypic changes also result from variation in these regions rather than in coding regions [2,81,82]. A relatively straightforward way to investigate this is to first analyze sequence variation and search for specific motifs in promoter regions [83]. When analyzing the *Asr2* promoter region, it is quite remarkable how low nucleotide diversity is especially in the Quicacha population. The observed polymorphism pattern suggests that a (incomplete) selective sweep eliminated nucleotide diversity at *pAsr2* and increased the frequency of one favored haplotype. This may indicate an important function of this region. Similarly, the downstream region of *pLC30-15* shows low nucleotide diversity and patterns consistent with positive selection in the Tacna population, which may also be functionally significant. Unlike *pAsr2* and *3’pLC*, *pAsr4* and *5’pLC* are more polymorphic than their corresponding coding regions. The patterns consistent with local adaptation at *Asr4* in the Tacna population described before [47] disappear at *pAsr4* and neutrality tests show no deviation from a standard neutral scenario. Also, the haplotype structure observed in Quicacha [47] is not present at *pAsr4*. This shows that the evidence for local adaptation described before is limited to the *Asr4* coding regions. On the other hand, the haplotype structure and patterns of positive selection at the *pLC30-15* locus described in [30] in Quicacha remain at *5’pLC*, suggesting that the acting selective forces are not limited to the gene. 

 Although we found evidence for positive selection in the coding regions of *Asr2*, *Asr4* and *pLC30-15* and also in some of their regulatory regions, it is difficult to establish a relationship between the sequence evolution of the genes and their expression profiles. Except for the possible effects of trans-regulatory elements, reasons for this may be that more samples from different environments, larger sample sizes and more genes need to be investigated. Nonetheless, our approach may prove useful as an initial step towards determining whether these genes are involved in the adaptation of wild tomatoes to abiotic factors such as drought and cold. Indeed, an encouraging sign might be that our motif scan analysis shows conservative regulatory elements that are involved in drought, cold, heat stress and general stress responses at *pAsr2*, *5’pLC* and *3’pLC*. Further evidence about the possible relationship between sequence and gene expression variation is discussed below, after reviewing the results from related studies. 

### Environment-specific gene expression regulation of stress-responsive genes and its possible relationship to sequence variation

*Asr* genes have already been described to be induced by desiccation in several plant species, e.g. *Solanum chacoense* (wild potato) [84], *Pinus taeda* (loblolly pine) [85], *Lilium longifolium* (lily) [86], *Ginkgo biloba* [87], and *O. sativa* [88], but also by cold in *S. tuberosum* [89]. *Asr* genes have been shown to be very variable in their expression kinetics. They show differences in organ-specific expression [43,85] and different patterns depending on the gene copy [44,88] and applied stress [87,89]. Philippe et al. [88] even demonstrate differential expression patterns of rice *Asr* genes depending on the cultivar. Such variability in gene expression between accessions was also described for cold-responsive genes in wild tomato [18]. Similarly, although they share the same environment, the *S. peruvianum* accessions from *Tarapaca* (LA2744 and LA2745) seem to show different expression patterns for *Asr2*, *Asr4* and *pLC30-15*. Therefore, differences in gene expression may not necessarily be adaptive. It has been demonstrated that stress-related genes have a more variable expression than housekeeping genes [90,91] and a higher expression divergence of duplicated genes in *A. thaliana* [92]. In tomato, we detected different expression patterns between populations from similar environments only if the genes were in general lowly expressed. This is in accordance with the findings of Carey et al. [93], who found that transcriptional noise decreases as the expression level increases. 

Although we cannot establish a clear connection between gene expression variation and patterns of diversity in the regulatory regions or the coding regions analyzed in previous studies [30,45-47], we discovered interesting trends. First, we suggest that the *Asr* genes and *pLC30-15* are more strongly induced by water deficit than by cold, indicating that they play a more important role in drought response than in cold response in wild tomato species. In accordance with the results by Carey et al. [93] discussed above, the expression patterns of the *Asr* genes and *pLC30-15* appear to be more noisy after cold stress. Second, *Asr1* seems to show a similar expression pattern after drought stress in all *S. chilense* and *S. peruvianum* populations, i.e. induction occurs after 1-3h and relative expression increases until 24h, while the expression level is lower and more noisy after cold stress. *Asr1* homologs have been shown to be conserved in wild tomato and other plant species and seem to act as housekeeping genes [44,47]. Our results suggest a concordant conserved expression pattern, highlighting the importance of *Asr1* for basic functions in the plants. Third, *Asr2* expression appears to be quite low compared to the other candidate genes. This might indicate that *Asr2* does not play a major role in drought and cold response, but rather in other stress conditions. Another possible explanation for the relatively low expression of *Asr2* is that it is predominantly expressed in other organs than leaves. Indeed, Maskin et al. [43] found *Asr2* to be up-regulated in roots – but not in leaves – of cultivated tomato after drought stress. As we do not discover a high expression in leaves, organ-specific expression of *Asr2* might also occur in wild tomato species. However, more tissues should be tested to validate these findings.

 Finally, under water deficit *pLC30-15* and *Asr4* are more quickly induced in the accession from Tacna (hyperarid habitat) than in the other *S. chilense* accession from Quicacha (less dry habitat). However, we also observe a down-regulation of *Asr4* in the accession from Tacna after 3h. This could explain previous findings [44], in which *Asr4* expression could not be detected after 24h in drought-stressed wild tomato plants from a dry environment. Induction and down-regulation are faster in populations from dry habitats and the transcript was not sufficiently abundant to be detected. In the Tacna population, Fischer et al. [47] described a predominant *Asr4* haplotype, which is absent in other *S. chilense* populations. Interestingly, the accession tested here is homozygous for this haplotype which further highlights the abundance of this haplotype in the Tacna population. In addition, after cold stress *Asr4* seems to be more strongly induced in the Tacna accession from a high altitude compared to the other *S. chilense* accessions. As for the other genes studied here, however, *Asr4* expression appears to be in general much lower and therefore tends to be noisier after cold treatment. 

## Conclusions

Wild relatives of crop species have many advantages that make them interesting resources for studying plant evolution. One of them is that they are sampled from the ecological context they evolved in. We analyzed the expression variation of candidate genes in natural wild tomato populations. Such a rare study gives insights into natural variation in gene expression and can provide good candidates for improving plant tolerance to abiotic stresses. We found *Asr4* to be an interesting candidate, in accordance with a previous study [47]. Our observations suggest that both *Asr2* and *Asr4* as well as *pLC30-15* are induced by abiotic stresses, particularly by drought. The present study, as well as some others carried out in wild tomatoes [30,45-47], indicate that a candidate gene approach is efficient for detecting evidence for local adaptation to abiotic stresses and that wild tomato species constitute a valuable genetic resource for genes conferring resistance to abiotic stress. The genome of cultivated tomato became recently available [94]. Therefore, the evolution of regulatory elements can now be analyzed much more comprehensively, as has been done in *Arabidopsis* [95]. Finally, our results that positive selection occurs more often in local *S. chilense* populations and gene expression responses appear to be generally faster and stronger in this species seem to support the previous conclusions that *S. chilense* shows more evidence of local adaptation to drought and temperature stress than *S. peruvianum* [30,47,96].

## Supporting Information

File S1 **Contains additional Figures and Tables: Table S1 Numbers of sequenced haplotypes.** Table **S2** Primer sequences and amplification details for PCR of *pAsr2*, *pAsr4*, *5’pLC*, and *3’pLC*. Table **S3** Primer sequences and amplification details for the qPCR of the *Asr* genes, *pLC30-15*, and the reference genes. Table **S4** Summary of function and sequences of motifs found at *pAsr2, pAsr4, 5'pLC*, and *3'pLC* using PlantCARE. Figure **S1**: Sliding window analysis of (A) π and (B) Tajima’s *D* for the Tacna (red) and the Quicacha (green) populations over *pAsr2*. The x-axis indicates the location relative to the start codon of the *Asr2* gene; purple boxes indicate regulatory motifs. Figure **S2**: Sliding window analysis of (A) π and (B) Fay & Wu’s *H* for the Tacna (red) and the Quicacha (green) populations over *3’pLC*. The x-axis indicates the location relative to the stop codon of the *pLC30-15* gene; purple boxes indicate regulatory motifs. Figure **S3**: *pAsr2* haplotypes for the Tacna and Quicacha populations. Only polymorphic sites are shown. The number behind the sequences indicates the frequency of each haplotype. Figure **S4**: *5’pLC* haplotypes for the Tacna and Quicacha populations. Only polymorphic sites are shown. The number behind the sequences indicates the frequency of each haplotype.
(PDF)

File S2 ***pAsr2* alignment**
(FAS)

File S3 ***pAsr4* alignment**
(FAS)

File S4 ***5’pLC* alignment**
(FAS)

File S5 ***3’pLC* alignment**
(FAS)
