# Supplementary material for: Sequence Evolution and Expression Regulation of Stress-Responsive Genes in Natural Populations of Wild Tomato
Source: PLoS One. 2013 Oct 18;8(10):e78182. doi: 10.1371/journal.pone.0078182 (PMC3799731; doi:10.1371/journal.pone.0078182)
Supplement: File S1 — Contains additional Figures and Tables: Table S1 Numbers of sequenced haplotypes. Table S2 Primer sequences and amplification details for PCR of pAsr2, pAsr4, 5’pLC, and 3’pLC. Table S3 Primer sequences and amplification details for the qPCR of the Asr genes, pLC30-15, and the reference genes. Table S4 Summary of function and sequences of motifs found at pAsr2, pAsr4, 5'pLC, and 3'pLC using PlantCARE. Figure S1: Sliding window analysis of (A) π and (B) Tajima’s D for the Tacna (red) and the Quicacha (green) populations over pAsr2. The x-axis indicates the location relative to the start codon of the Asr2 gene; purple boxes indicate regulatory motifs. Figure S2: Sliding window analysis of (A) π and (B) Fay & Wu’s H for the Tacna (red) and the Quicacha (green) populations over 3’pLC. The x-axis indicates the location relative to the stop codon of the pLC30-15 gene; purple boxes indicate regulatory motifs. Figure S3: pAsr2 haplotypes for the Tacna and Quicacha populations. Only polymorphic sites are shown. The number behind the sequences indicates the frequency of each haplotype. Figure S4: 5’pLC haplotypes for the Tacna and Quicacha populations. Only polymorphic sites are shown. The number behind the sequences indicates the frequency of each haplotype. (PDF) [file pone.0078182.s001.pdf]

Supplementary Material

Sequence evolution and expression regulation of stress-responsive genes in natural populations of wild tomato

Iris Fischer, Kim A. Steige, Wolfgang Stephan and Mamadou Mboup

Table S1 Numbers of sequenced haplotypes

Species/populations	*pAsr2*	*pAsr4*	*5'pLC*	*3'pLC*
S. chilense	13	21	19	20
Quicacha	7	10	7	9
Tacna	6	11	12	11
S. peruvianum	19	16	19	19
Canta	11	9	10	9
Tarapaca	8	7	9	10

Table S2 Primer sequences and amplification details for PCR of *pAsr2*, *pAsr4*, *5'pLC*, *3'pLC*

locus	type of primer	primer sequence 5' → 3'	annealing temperature	location of last base*
pAsr2	forward	CTG GTG ACA ACT TCT ATG AGG	60°C	65261
	reverse	AGA TCG CTG TGG TGC TTC	60°C	66708
pAsr4	forward	TAG TAA ACG CGT ATT GAT GTG	61°C	102970
	reverse	CCC AAG TTC TTC AAG ATG C	61°C	104460
5'pLC	forward	CGT CAA ACC GAC CAC CTG	58°C	64005
	reverse	GGG ATG AAC GAA AGA GAG AC	58°C	62481
3'pLC	forward	GAA GTG GAA CAC AAG GAG GAG	58°C	62545
	reverse	CAA GTT GTG ACG CCA TAC C	58°C	60005

*Location of primers for *Asr* upstream regions according to GenBank accession CU468249; of *pLC30-15* flanking regions according to accession CU041361.4.

Table S3 Primer sequences and amplification details for the qPCR of the *Asr* genes, *pLC30-15*, and the reference genes

Locus	Type of primer	primer sequence 5' → 3'	annealing temperature	location of last base*
Asr1	forward	CAA ATC GGT AAA CTT GGC A	55°C	96967
	reverse	TGG TGT CCC CCC TCA G	55°C	97833
Asr2	forward	CAC CAT CAC CAT TTG TTC C	55°C	66655
	reverse	CTT CTT TGC CTT GTG TTT CTC	55°C	66890
Asr4	forward	TGG TGG AGG AGT TGG TG	65°C	104348
	reverse	TTG TGT GCA TGC TCT GGA	65°C	104635
pLC30-15	forward	AAG TGG AAC ACA AGG AGG AG	59°C	62545
	reverse	ATC TTC TGT CCA TCC TCT CCA	59°C	62246
CT189	forward	GGG TTC CGA AGA ATC TAT	58°C	674
	reverse	GTT GAA GAA TGT GGC GTG	58°C	736
TIP4I	forward	ATG GAG TTT TTG AGT CTT CTG C	63°C	see [1]
	reverse	GCT GCG TTT CTG GCT TAG	63°C	see [1]

*Location of primers for *Asr* genes according GenBank accession CU468249; of *pLC30-15* according accession CU041361.4, of CT189 according to accession DQ104676.1. But note that qPCR primers were designed on conserved regions in *S. chilense* and *S. peruvianum* alignments, except for *TIP4I* which was taken from [1].

Table S4 Summary of function and sequences of motifs found at *pAsr2, pAsr4, 5'pLC*, and *3'pLC* using PlantCARE.

Motif	Function	Sequence	found at
ABRE	*cis*-acting element involved in the abscisic acid responsiveness	CGTACGTGCA	*pAsr4, 5'pLC, 3'pLC*
ARE	*cis*-acting regulatory element essential for the anaerobic induction	TGGTTT	*pAsr2, pAsr4*
AuxRR-core	*cis*-acting regulatory element involved in auxin responsiveness	GGTCCAT	*pAsr4, 3'pLC*
CGTCA-motif	*cis*-acting regulatory element involved in methyl jasmonate responsiveness	CGTCA	*5'pLC, 3'pLC*
ERE	ethylene-responsive element	ATTTCAAA	*pAsr2, pAsr4*
HSE	*cis*-acting element involved in heat stress responsiveness	AAAAAATTTC	*3'pLC*
LTR	*cis*-acting element involved in low-temperature responsiveness	CCGAAA	*3'pLC*
MBS	MYB (transcription factor) binding site involved in drought-inducibility	C/TAACTG	*pAsr2, 5'pLC, 3'pLC*
TC-rich repeats	*cis*-acting element involved in defense and stress responsiveness	ATTTTCTTCA	*pAsr2, 5'pLC, 3'pLC*
TCA-element	*cis*-acting element involved in salicylic acid responsiveness	CCATCTTTTT	*pAsr2*

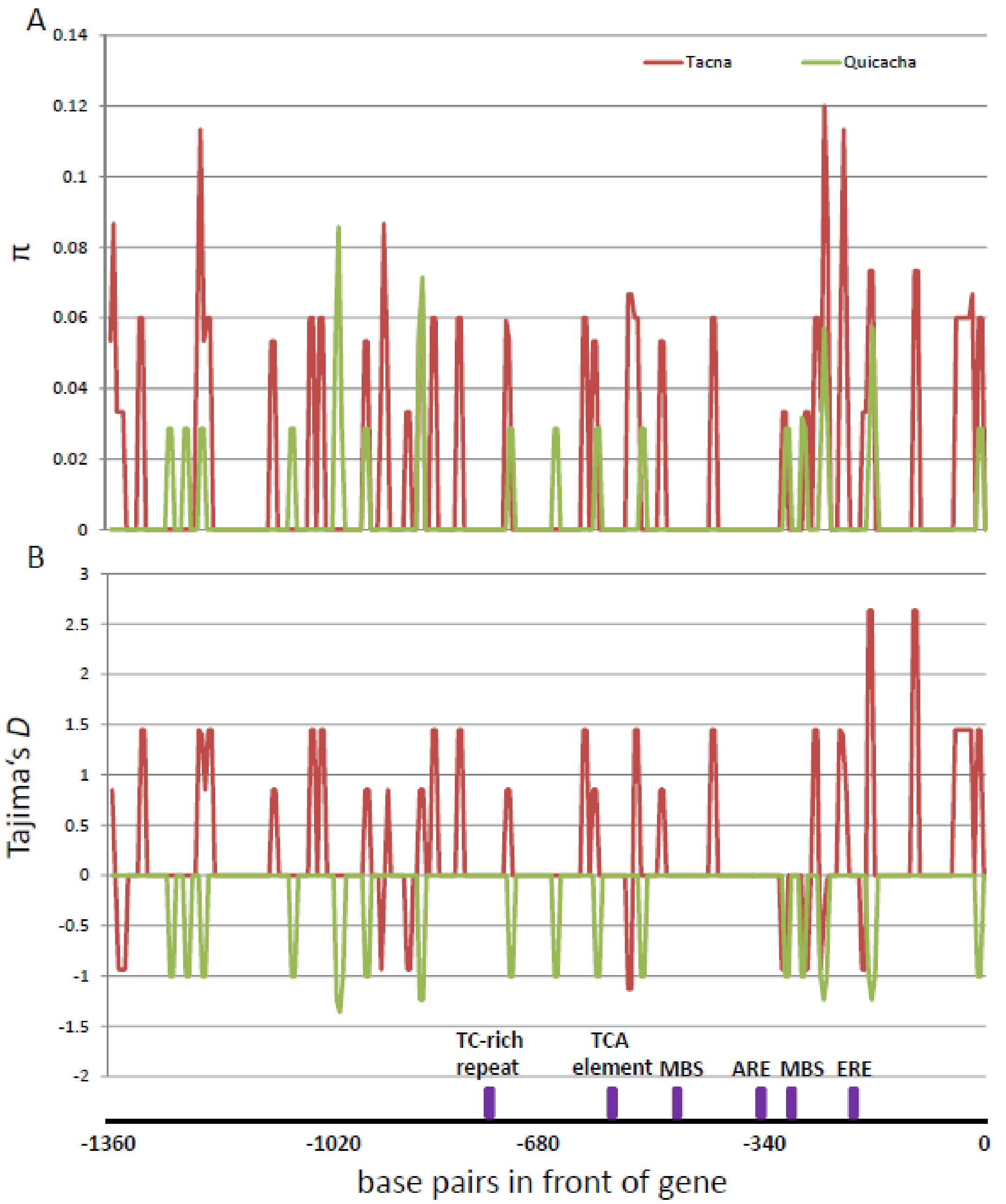

Figure S1: Sliding window analysis of (A) π and (B) Tajima's *D* for the Tacna (red) and the Quicacha (green) populations over *pAsr2*. The x-axis indicates the location relative to the start codon of the *Asr2* gene; purple boxes indicate regulatory motifs.

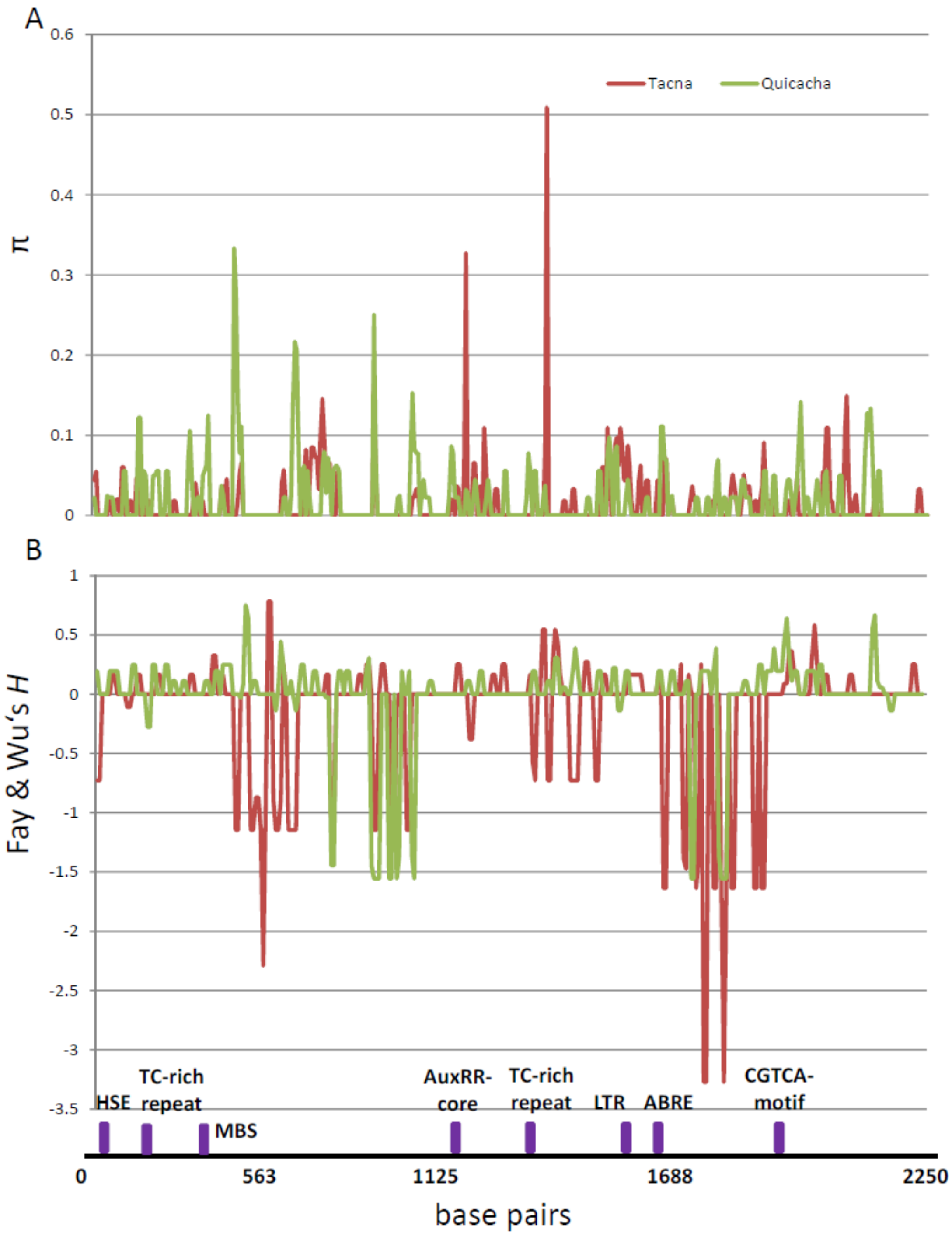

Figure S2: Sliding window analysis of (A) π and (B) Fay & Wu's *H* for the Tacna (red) and the Quicacha (green) populations over *3'pLC*. The x-axis indicates the location relative to the stop codon of the *pLC30-15* gene; purple boxes indicate regulatory motifs.

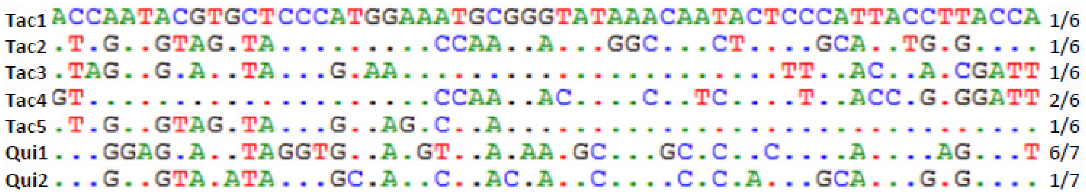

Figure S3: *pAsr2* haplotypes for the Tacna and Quicacha populations. Only polymorphic sites are shown. The number behind the sequences indicates the frequency of each haplotype.

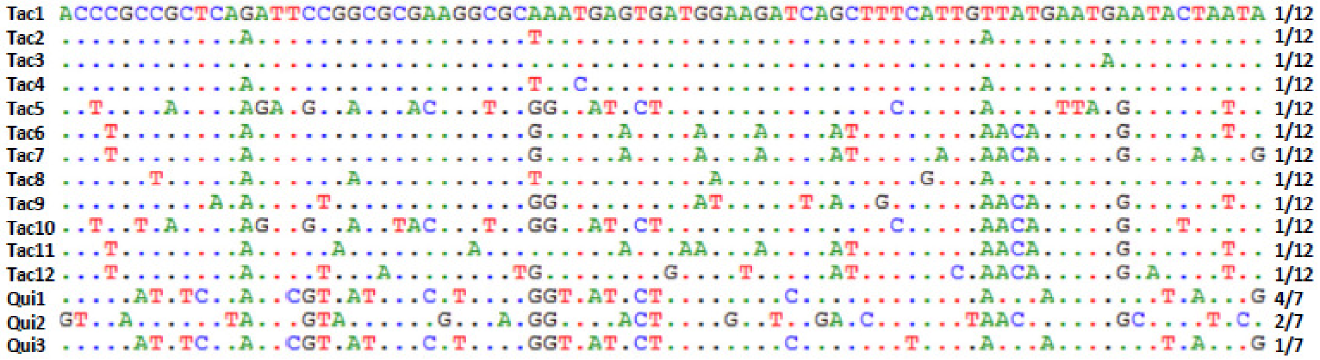

Figure S4: *5'pLC* haplotypes for the Tacna and Quicacha populations. Only polymorphic sites are shown. The number behind the sequences indicates the frequency of each haplotype.

Reference:

1. Exposito-Rodriguez M, Borges AA, Borges-Perez A, Perez JA (2008) Selection of internal control genes for quantitative real-time RT-PCR studies during tomato development process. BMC Plant Biol 8: 131.
